# Effects of *trans*-2-hexenal and *cis*-3-hexenal on post-harvest strawberry

**DOI:** 10.1038/s41598-019-46307-4

**Published:** 2019-07-12

**Authors:** Junko Wakai, Shoko Kusama, Kosuke Nakajima, Shikiho Kawai, Yasuaki Okumura, Kaori Shiojiri

**Affiliations:** 1 Panasonic Corporation, Technology Innovation Division, 3-4 Hikaridai, Seika-cho, Soraku-gun, Kyoto, Japan; 2 Department of Agriculture, Ryukoku University, 1-5 Yokotani, Seta oe-cho, Otsu, Shiga Japan

**Keywords:** Abiotic, Jasmonic acid, Plant physiology

## Abstract

Green leaf volatiles are emitted by green plants and induce defence responses. Those with antifungal activities in plants may replace chemicals as natural post-harvest treatments. We investigated the postharvest treatment of strawberry with *trans*-2-hexenal and *cis*-3-hexenal and observed a decrease in the mould infection rate. To determine the volatiles’ functions, we conducted a component analysis of the volatiles released from *trans*-2-hexenal-treated strawberry and analysed gene expression. Several acetates, which were expected to be metabolites of *trans*-2-hexenal in fruit, were released from treated strawberry; however, these acetates did not inhibit fungal growth. The gene expression analysis suggested that postharvest strawberries were not protected by jasmonic acid-mediated signalling but by another stress-related protein. Harvested strawberries experience stress induced by harvest-related injuries and are unable to perform photosynthesis, which might result in different responses than in normal plants.

## Introduction

Intra- and inter-plant signalling events in a variety of plants involve volatile organic compounds (VOCs). Damaged plants release VOCs, such as green leaf volatiles (GLVs) and terpenoids. The surrounding intact plants that are exposed to the VOCs exhibit defence-related responses before any damage occurs^1–5^. Recombinant *Arabidopsis thaliana* having an enhanced lipoxygenase activity, which is related to the GLV biosynthetic pathway, releases *cis*-3-hexenal upon predation by *Pieris rapae* larvae or infection by *Botrytis cinerea*, and displays an increased tolerance level^6^. GLVs include the aldehydes, esters, and alcohols of six-carbon compounds. In the GLV biosynthetic pathway, *cis*-3-hexenal is formed from linolenic acid by various enzymatic reactions and is converted to alcohol through a trans-2-hexenal via an isomerase reaction^5^. Subsequently, acetate is generated by an alcohol acyl CoA transferase reaction. Among GLVs, exposure of *A. thaliana* to C6 aldehydes, such as *trans*-2-hexenal and *cis*-3-hexenal, induces the expression of genes involved in biological defence and resistance to grey mould^7,8^. In plants, GLVs, especially C6 aldehyde, which is thought to be a strong inducer, are involved in biological defence responses. The enhanced immune functions induced by GLVs in plants have the potential to replace agricultural chemicals as natural plant defences. Studies on GLVs have investigated mainly living plants, and only a few studies have targeted post-harvest crops.

To prevent the postharvest infection of vegetables and fruits by pathogens, such bacteria and fungi, chemical compounds have been used. These methods are problematic because of chemical contamination, residuals, and degradability. Thus, the use of natural compounds, such as GLVs, as postharvest treatments has been investigated. GLVs have been used as antifungal fumigation agents, and hexanal reduces the decay of apple^9^, raspberry, peach^10^, and pear^11^. GLVs function as antifungal agents, and may also induce immune responses in fruit and vegetables after harvest. Tomatoes treated with *trans*-2-hexenal have decreased *B. cinerea* infection rates and increased expression levels of ethylene receptors, lipoxygenase, and phenylalanine ammonia lyase compared with those treated with distilled water^12^. Thus, jasmonic acid (JA)/ethylene-induced systemic resistance may be involved in the reduced infection rate observed in those studies. Interestingly, living and post-harvested plants demonstrate similar defence responses, which are induced by C6 aldehyde. The induction of mould resistance in strawberries would provide an effective post-harvest treatment, because losses owing to mould damage during transport are high. However, there are limitations associated with the use of C6 aldehyde. High concentrations of *trans*-2-hexenal directly inhibit fungal growth but are associated with the induction of adverse events in plants^13^. At low concentrations, *trans*-2-hexenal may promote hyphal growth^14^. Therefore, the use of GLVs on harvested strawberry fruit is complicated. Because C6 aldehydes, such as *trans*-2-hexenal and *cis*-3-hexenal, induce the expression of defence genes, it may be possible to induce tolerance against fungi at a considerably lower concentration than reported in previous studies that investigated direct antifungal activities.

In this study, we investigated the optimal concentrations the of C6 aldehydes *trans*-2-hexenal and *cis*-3-hexenal as postharvest treatments of strawberry to decrease the *B. cinerea* infection rate. To determine how *trans*-2-hexenal induces an antifungal effect against *B. cinerea*, volatiles from treated strawberries were analysed and a gene expression analysis was performed.

## Results

### Effect of GLVs on *B. cinerea* infection in strawberry

At 24 h after the GLV treatments, except 50-µmol (420 nmol/cm^3^) *trans*-2-hexenal, no colour changes were observed in strawberries, (Supplementary Data 1). Strawberry necrosis occurred following treatment with 50-µmol *trans*-2-hexenal (Supplementary Data 2). Necrosis induced by *trans*-2-hexenal had been reported previously^13^. Therefore, strawberries treated with 50-µmol *trans*-2-hexenal were not used for further study. A strawberry serving as a host of *B. cinerea* hyphal expansion is shown in Supplementary Data 3.

**Figure 1:**
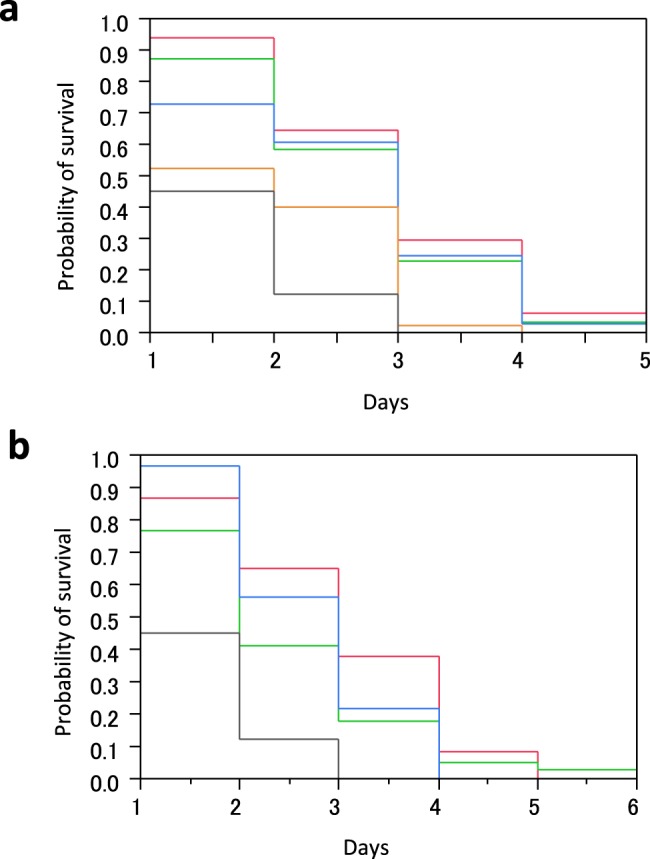
Survival analysis of strawberry following *B. cinerea* infection. The number of infected strawberries and the date of infection were recorded. A survival analysis was performed using JMP7.0.2. (**a**) *cis*-3-hexenal and (**b**) *trans*-2-hexenal treatments are shown. The red, green, blue, orange, and black lines represent treatments of 0.1, 1.0, 5.0, 50.0, and 0 µmol, respectively. For each concentration of hexenal, 0.1 µmol was 0.8 nmol/cm^3^, 1.0 µmol was 8 nmol/cm^3^, and 5.0 µmol was 42 nmol/cm^3^. The day when *B. cinerea* was brought into contact with the volatile-treated strawberry was regarded as d 0. The concentrations represent those of the volatile constituents processed in the strawberry.

Survival curves were similar between *cis*-3-hexenal- and *trans*-2-hexenal-treated strawberries (Fig. 1a,b). The survival rates of strawberries treated with volatiles were greater than that of the control (*trans*-2-hexenal: df = 3, P < 0.001; *cis*-3-hexenal: df = 4, P < 0.001). Approximately 60% of strawberries treated with 0.1-µmol (0.8 nmol/cm^3^), 1-µmol (8 nmol/cm^3^), and 5-µmol (42 nmol/cm^3^) *cis*-3-hexenal survived after 2 d, and ~40% of those treated with 50-µmol (420 nmol/cm3) *cis*-3-hexenal survived after 1 d, with the day *B. cinerea* was placed on fruit being d 0. However, only ~10% of the control strawberries survived (Fig. 1a). Additionally, ~60% of strawberries treated with 0.1-µmol (0.8 nmol/cm^3^) and 1-µmol (8 nmol/cm^3^) - *trans*-2-hexenal, and ~40% treated with 5-µmol (42 nmol/cm^3^) *trans*-2-hexenal, survived after 2 d. After 3 d, all the control strawberries were infected, and some of those treated with *trans*-2-hexenal survived for 5 d (Fig. 1b). Among the volatile concentrations, the effects of 0.1-µmol *trans*-2-hexenal and *cis*-3-hexenal were greatest. In a subsequent analysis, strawberries treated with 1-µmol (8 nmol/cm^3^) *trans*-2-hexenal had the longest survival time.

### Volatiles from GLV-treated strawberries

**Figure 2:**
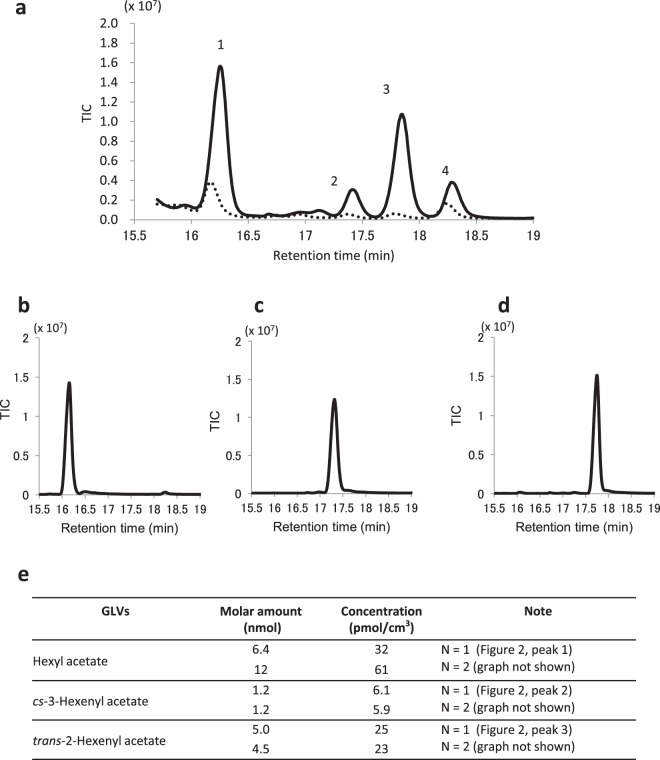
Analysis of volatiles from strawberry treated with *trans*-2-hexenal. Gas components released from strawberry treated with *trans*-2-hexenal or hexane were analysed. Hexane was used as the control. (**a**) The total ion chromatograph (TIC) of gas from strawberry is shown at a retention time of ~17 min. The solid line represents the gas components generated by the *trans*-2-hexenal treatment, and the dotted line represents the gas components generated by the hexane treatment. Peak 1 is hexyl acetate, peak 2 is *cis*-3-hexenyl acetate, and peak 3 is trans-2-hexenyl acetate. Peak 4 at the retention time of ~18.3 min was also relatively high in the control. Therefore, peak 4 was not analysed; (**b**) TIC of 1 nL standard hexyl acetate reagent; (**c**) TIC of 1 nL standard cis-3-hexenyl acetate reagent; (**d**) TIC of 1 nL standard trans-2-hexenyl acetate reagent; (**e**) The molar amount of each GLV was measured by calibration using standard reagents. The concentration was calculated by the gas sampling volume (200 mL). Volatiles from GLV-treated strawberries were analysed twice.

Volatiles released from *trans*-2-hexenal-treated strawberries were collected and analysed using gas chromatography–mass spectrometry (GC–MS). We examined a total ion chromatograph with a retention time of 16–19 min because there were differences between treated and control strawberries. Interestingly, *trans*-2-hexenal was not detected. Thus, the added odour did not remain on the strawberries. It was hypothesized that *trans*-2-hexenal did come into contact with *B. cinerea*. Four peaks were detected from the treated strawberries. Peak 1 was hexyl acetate, peak 2 was *cis*-3-hexenyl acetate, and peak 3 was *trans*-2-hexenyl acetate (Fig. 2a). Figure 2b–d show chromatographs of commercially available reagents of these acetates, respectively. Peak 4 occurred at a retention time of 18.3 min and was predicted to be 2-hexenoic acid ethyl ester or 1-hexenol; however, it was also detected at a relatively high level in the control, with height of the peak being ~50% that in the *trans*-2-hexenal treatment. Therefore, peak 4 was not identified nor described further. The concentration of each acetate (hexyl acetate, *cis*-3-hexenyl acetate, and *trans*-2-hexenyl acetate) was low following calibration and was in the order of pmol/cm^3^ (Fig. 2e).

### Effect of GLVs on the growth of *B. cinerea*

**Figure 3:**
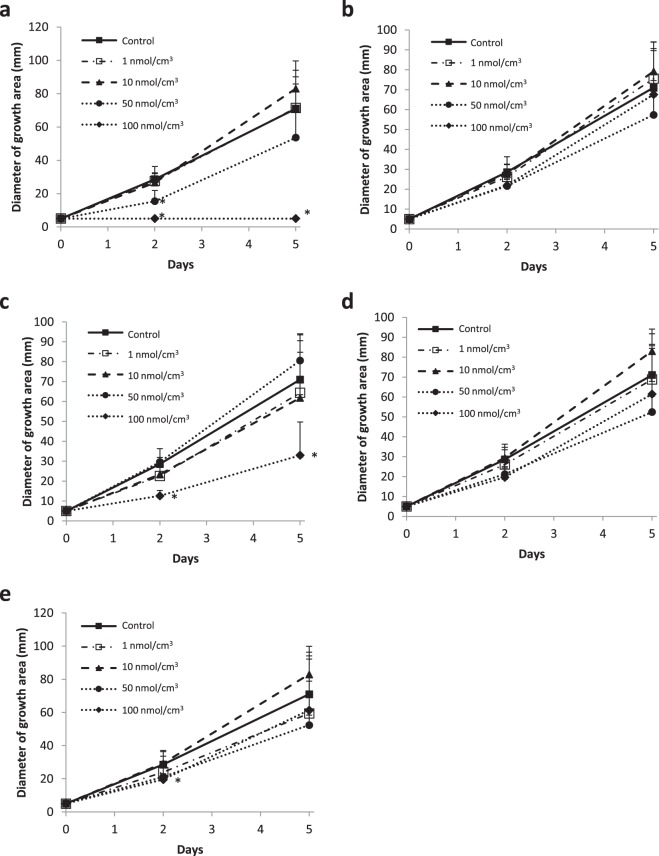
The growth of *B. cinerea* in the presence of green leaf volatiles (GLVs). *B. cinerea* was incubated on PDA medium with a GLV gas at 25 °C. After 2 or 5 d, the diameters of the growth areas were measured. The averages of the PDA plates (N = 5) are plotted, and the error bars represent standard deviations. The GLVs used were (**a**) *tran*s-2-hexenal; (**b**) *cis*-3-hexenal; (**c**) *trans*-2-hexenyl acetate; (**d**) *cis*-3-hexenyl acetate; and (**e**) hexyl acetate. The GLV concentrations were 1, 10, 50, and 100 nmol/cm^3^. As a control, hexane, which was the diluting solvent, was used. The PDA agar plates had 85-mm diameters.

To determine whether the volatile components released from strawberry inhibited *B. cinerea*’s growth, the growth of mould in the presence of acetate was observed *in vitro*. For comparison, *cis*-3-hexenal and *trans*-2-hexenal treatments were also tested on mould. The concentrations of these GLVs were 0.001 to 100 nmol/cm^3^, including those of GLVs that had been used to treat the strawberries. As the day increased, the fungal area expanded (Fig. 3a–e). When incubated in the presence of high concentrations of *trans*-2-hexenal, *trans*-2-hexenyl acetate, and hexyl acetate, mycelial growth was inhibited compared with that of the control. At GLV concentrations of 10 nmol/cm^3^ or less, hyphal growth was not inhibited.

### Gene expression analysis

A gene expression analysis using next-generation sequencing (RNA-seq) was performed by integrating data from 10 *trans*-2-hexenal-treated strawberries. The read sequences were mapped to the genome, and expression data of 14,593 genes were obtained. Log 2 values of the fragments per kilobase of transcript per million mapped reads (FPKMs) were calculated to normalize the counts. The quality of transcriptome sequencing can be found in Supplementary Data 4. The SRA number is PRJNA526582.

**Figure 4:**
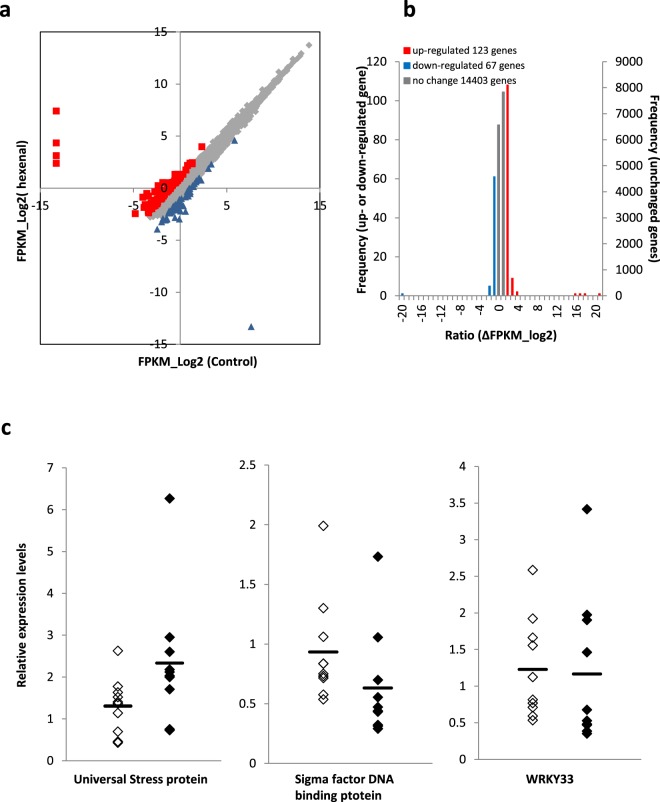
Gene expression in *trans*-2-hexenal-treated strawberry. (**a**) The expression levels of the genes following volatile treatment are shown. Red squares indicate genes with expression ratios of hexenal treatment to control of 1 or more, representing up-regulation. Blue triangles indicate an expression ratio of −1 or less, representing down-regulation. Grey diamonds indicate genes with expression ratios between 1 and −1, representing no change in the expression level. Plots in the vicinity of −13 on the X- or Y-axis indicate a gene with an expression level of 0; (**b**) A histogram of the genes is shown. The X-axis represents the expression ratio; (**c**) The expression levels of three genes in each of 10 individual strawberries were plotted. An analysis was performed separately using real-time PCR. The elongation factor 1-alpha gene was used as the internal standard. The expression levels of genes were determined using a relative standard curve. Filled diamonds indicate *trans*-2-hexenal-treated samples, open diamonds represent controls, and the bars represent average values.

Then, log expression levels were compared with those of the controls. In total, 123 genes were up-regulated (red squares in Fig. 4a), and 67 genes were down-regulated (blue triangles in Fig. 4a). Overall, the changes in expression owing to the presence or absence of *trans*-2-hexenal treatment were small (Fig. 4a).

The expression levels of defence-related genes, such as those in the GLV biosynthetic pathway, were not up-regulated. Although there were few changes, two genes with comparatively high expression levels after the *trans*-2-hexenal treatment were selected. Sigma factor-binding protein 1 chloroplastic-like and universal stress protein A-like protein were selected as genes with greater expression levels than in the control. The expression levels of these two genes and WRKY33, which is predicted to be associated with sigma factor-binding protein 1 chloroplastic-like, in 10 strawberries were analysed using real-time PCR (Fig. 4c). Compared with the control, there were no differences in the expression levels of sigma factor-binding protein 1 (t-test, P = 0.14) and WRKY 33 (t-test, P = 0.87). The expression of universal stress protein A-like protein was slightly up-regulated; however, because of individual differences among strawberry samples the t-test value was P = 0.07. An additional 19 potentially up-regulated genes were analysed, but significant up-regulation was not confirmed (Supplementary Data 5).

### Jasmonic acid (JA) analysis

**Figure 5:**
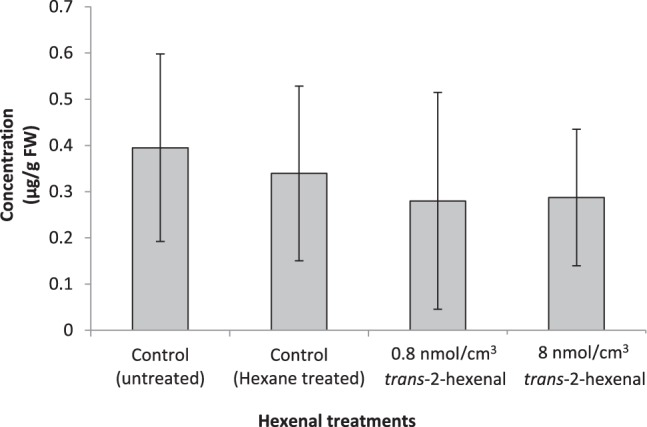
Quantification of jasmonic acid in strawberry. The concentration of jasmonic acid in *trans*-2-hexenal-treated strawberry is shown. The hexenal treatments were 0.8 nmol/cm^3^ and 8.0 nmol/cm^3^. As controls, hexane-treated and untreated strawberries were used. Bruised strawberries were excluded from the analysis. The numbers of strawberries used for the analysis were 9, 6, 8, and 6 for untreated, hexane, 0.8 nmol/cm^3^ of *trans*-2-hexenal and 8.0 nmol/cm^3^ of *trans*-2-hexenal. The data in the graph are averages, and error bars represent the standard deviations. FW, frozen weight of strawberry fruit.

There were no differences in JA levels among untreated, hexane-treated, and *trans*-2-hexenal-treated strawberries (Fig. 5). The different treatments did not affect the JA concentration.

## Discussion

Exposure to *trans*-2-hexenal or *cis*-3-hexenal acted as an effective postharvest treatment to decrease infection by *B. cinerea*. A 0.1-µmol concentration (0.8 nmol/cm^3^) of either agent was sufficient to induce tolerance, and this value was lower than the concentration that induces tissue necrosis. The same experiment was repeated twice on separate days, and similar results were obtained. In different strawberry varieties, similar trends were found but the duration of the antifungal effect varied (data not shown). Strawberry condition may be an important factor for effective antifungal treatments.

The strawberries exposed to *trans*-2-hexenal released hexyl acetate, *trans*-2-hexenyl acetate, and *cis*-3-hexenyl acetate. Trans-2-hexenal was not detected. In this experiment, strawberries exposed to *trans*-2-hexenal for 1 d were then washed with distilled water, transferred to a new plastic cup, and infected with mould. The acetate concentration released from strawberries was low and did not inhibit fungal growth. The amount of *trans*-2-hexenal used was not sufficient to inhibit mould growth. Therefore, the gas component released from strawberries following *trans*-2-hexenal treatment did not contribute to the plant’s defence against fungal infection. Mould resistance on strawberries was not a direct effect of GLVs. However, the reason for the acetate release is of interest. In the GLV biosynthetic pathway, *cis*-3-hexenal is formed from linolenic acid by various enzymatic reactions and is converted to alcohol by *trans2*-hexenal through an isomerase reaction^5^. Subsequently, acetate is generated by an alcohol acyl CoA transferase reaction. The acetates detected by GC–MS may result from *trans*-2-hexenal being taken into plant cells and metabolized by the GLV biosynthetic pathway, owing to the large amount of *trans*-2-hexenal entering the cell. Alternatively, the production of GLVs may increase following stimulation by hexanal.

The trans-2-hexenal taken into the strawberry may inhibit the growth fungi. C6 volatiles and their corresponding glycosides also play important roles in improving plant resistance^15^. C6 volatiles can be taken in and converted to glycosides in both tea^16^ and tomato^17^ plants. The glucosidase reaction induced by bacterial infection may generate volatiles from glycosides and cause antifungal resistant. The same effect may occur in strawberries treated with *trans*-2-hexenal. However, the lowest concentration of 0.1 µmol (0.8 nmol/cm^3^) induced sufficient resistance to *B. cinerea* in this experiment. Therefore, we predicted that another factor plays an important role.

A comprehensive gene expression analysis of hexenal-treated strawberries was performed. Changes in the expression levels of defence genes related to GLVs synthesis were not confirmed. Additionally, the amount of JA measured was not different between strawberries treated with *trans*-2-hexenal and hexane (Fig. 5). GLV synthesis may not have been activated.

In the present study, sigma factor-binding protein 1 chloroplastic-like and universal stress protein A-like protein were up-regulated compared with the control. In *Arabidopsis*, sigma factor-binding protein 1 is involved in salicylate- and JA-mediated defence responses^18^. The product of this gene activates WRKY33 and is strongly induced by grey mould^19^. In *Arabidopsis*, WRKY 33 is also a key regulator of protective responses against grey mould infection^20,21^. The expression levels of these genes were analysed using real-time PCR, but no increases were observed following hexenal treatments (Fig. 4c). Another selected gene, the universal stress protein gene, encodes a protein that responds to diverse abiotic stresses, such as fever, hydrogen peroxide, salt, drought, and low temperature^22–26^. The universal stress protein isolated from *Arabidopsis* has an antifungal activity^27^. Here, the expression of the universal stress protein A-like protein was slightly increased (Fig. 4c). A universal stress protein A-like protein has not been identified in strawberry; however, as reported in *Arabidopsis*, such as protein may be involved in the suppression of grey mould infection. GLV synthesis and pathogen-induced defences are basic functions shared by all plant species. However, our results obtained with strawberry fruit revealed that GLV synthesis and pathogen-induced defences were not functioning. Such differences may be the result of using harvested strawberries. The effects of strong injury-related stress at harvest and the inability to perform photosynthesis-related activities might result in different biological responses from those occurring in normal plants. If the amount of water is reduced as a result of stress at harvest, the universal stress protein may be induced, like under drought conditions. Under water-limiting conditions, photosynthetic carbon and energy metabolism are reduced, resulting in energy conservation^28^. Plants exposed to external stimuli, including GLVs, usually respond somewhat, such as with GLV production. However, this reaction may be limited in harvested fruit under energy-saving conditions. In fact, there were few marked changes in gene expression between *trans*-2-hexenal-treated and control strawberries. Additionally, changes in gene expression related to GLV production were small (Fig. 4a). Epigenetic modifications in the genomic region harbouring the universal stress protein may occur in harvested strawberry. The resistance of strawberry to grey mould following GLV treatments is likely caused by the secondary induction of the universal stress protein gene, which is activated at harvest, through a priming effect. These hypotheses are still unconfirmed and further analyses are needed in the future.

In summary, this study showed that strawberries can acquire resistance to fungi following post-harvest treatments with hexenal. Furthermore, this resistance did not result from the direct effects of hexenal and hexenyl acetate, but may involve antifungal-related genes. These resistance genes are not related to GLV biosynthesis or JA-mediated defence pathways, as previously reported. Strong injury stress at harvest may affect metabolism. To induce a better effect, it is important to consider the concentration of GLV, the type of strawberry, the number of days after harvesting, and detailed conditions. Studies on physiological differences between plants capable of photosynthesis and fruit incapable of energy production are rare. Further knowledge may lead to the development of preserved vegetables and fruit with well-balanced disease resistance and storage capabilities. This would be an important contribution to the problem of food waste.

## Methods

### Food materials and pathogen

Strawberries (*Fragaria* × *ananassa*, ‘Sagahonoka’) were purchased from a market in Japan. Fruit was sterilized by washing with detergent and a 70% ethanol spray. A *B. cinerea* strain was provided by Gifu University River Basin Research Center. Potato dextrose agar (PDA, Difco Laboratories, Detroit, MI, USA) was inoculated with the strain, which was then grown at 25 °C until confluent.

### GLV treatment of strawberry and *B. cinerea* infection

*trans*-2-Hexenal was purchased from Tokyo Chemical Industry Co., LTD. (Tokyo, Japan). *cis*-3-Hexenal was purchased from Santa Cruz Biotechnology, Inc. (Dallas, TX, USA). These volatiles were diluted with hexane (Fujifilm Wako Pure Chemical Co., Osaka, Japan) to 0.1, 1.0, and 5.0 M. A piece of filter paper and a strawberry sterilized by the method described above were placed in a 120-mL polyethylene terephthalate cup. After 1 µL of *trans*-2-hexenal or *cis*-3-hexenal solution was dropped on the filter paper, the plastic cup was immediately closed. As a result, one strawberry and 0.1, 1.0, or 5.0 μmol of volatile were enclosed in each 120-mL plastic cup. For each concentration of volatile, 0.1 μmol was 0.8 nmol/cm^3^, 1.0 μmol was 8 nmol/cm^3^, and 5.0 μmol was 42 nmol/cm^3^. Hexane (1 µL) was used as a control. Twenty strawberries were prepared per volatile concentration. The cup was incubated under 12-h/12-h light/dark conditions at 20 °C. After 24 h, the strawberries were removed from the cups and washed with detergent and a 70% ethanol spray. Any strawberries in poor condition were not used for subsequent experiments. Each strawberry was placed in a new cup, and then a 20-mm^2^ piece of agar on which *B. cinerea* was grown was placed on the fruit. The closed cup was incubated in the dark at 25 °C. The number of days before strawberry fruit decayed owing to hyphal expansion was recorded. The same experiment was repeated twice on other days. Because the survival time of the control hexane-treated strawberry was not determined prior to the experiment (df = 1, P = 0.23 by Wilcoxon test), data from the 2-d time point were integrated, and a survival analysis was performed using JMP7.0.2 (SAS Institute Inc., Cary, NC, USA) for each volatile. Bruised strawberries were excluded from the analysis. The numbers of strawberries used for the analysis were 34, 31, 33, and 40 for 0.1, 1.0, 5.0, and 50.0-µmol *cis*-3-hexenal, respectively, and 37, 39, and 32 for 0.1, 1.0, and 5.0-µmol *trans*-2-hexenal, respectively. Control hexane was used on 40 strawberries.

### Analysis of volatiles from GLV-treated strawberries

In the manner described above, strawberries and 1 μmol *trans*-2-hexenal were incubated in 120-mL plastic cups for 24 h. Hexane was used as a control. After washing with detergent and a 70% ethanol spray, fruit were placed in a clear sampling bag (GL Sciences Inc., Tokyo, Japan). The bag was left standing for 2 h at room temperature. A Tenax TA Adsorbent Resin (60/80) (GERSTEL GmbH & Co., Mülheim an der Ruhr, Germany) was attached to the sampling bag, and 200 mL of the gas in the bag was collected by pulling the suction pump. Tenax TA-absorbed gas was heat-desorbed using TDS/CIS (GERSTEL GmbH & Co.). Collected gas was analysed using an Agilent 6890/5973 A GC–MS system (Agilent Technologies, Inc., Santa Clara, CA, USA). An InertCap Pure-WAX column (GL Sciences Inc.) was used. The temperature was increased from 50 °C to 180 °C at a rate of 5 °C per min, and then to 250 °C at a rate of 20 °C per min. The temperature was held at 250 °C for 10 min. The ionization voltage was 70 eV. The *m/z* was measured from 35 to 400. Obtained data were analysed using Agilent Masshunter Qualitative Analysis B.07.00 software. *trans*-2-Hexenyl acetate, *cis*-3-hexenyl acetate, and hexyl acetate were purchased from Tokyo Chemical Industry Co., LTD.

### The growth of *B. cinerea* in the presence of GLVs

After *B. cinerea* was transplanted onto PDA medium in a plate, a filter paper was placed on the lid and a drop of GLV dissolved in hexane was placed on the paper. The GLVs used were *tran*s-2-hexenal, *cis*-3-hexenal, *trans*-2-hexenyl acetate, *cis*-3-hexenyl acetate, and hexyl acetate. The GLV concentrations were 1, 10, 50, and 100 nmol/cm^3^. As a control, hexane, which was the diluting solvent, was used. Then, the lid was quickly closed, and the plate was incubated at 25 °C. After 2 or 5 d, the diameters of the growth areas were measured. The PDA agar plates had 85-mm diameters.

### Expression analysis (RNA-seq)

In the manner described above, strawberries and 1 μmol *trans*-2-hexenal were incubated in 120-mL plastic cups for 24 h. Hexane was used as a control. Total RNA was purified using an ISOSPIN Plant RNA kit (Nippon Gene., LTD., Tokyo, Japan) from 10 strawberries treated with *trans*-2-hexenal according to the kit’s protocol. Subsequent processing and RNA-seq analyses were performed using a next-generation sequencer by TaKaRa Bio Inc. (Shiga, Japan). A sequence library was prepared from total RNA using a TruSeq Stranded mRNA Sample Prep Kit (Illumina, Inc., San Diego, CA, USA), according to the kit’s preparation guide (Rev. E). PolyA+ RNA was isolated from total RNA and fragmented. Then, single-stranded cDNA was synthesized by reverse transcription using fragmented RNA as the template. Using this single-stranded cDNA as the template, double-stranded cDNA incorporating dUTP was synthesized. After smoothing and phosphorylating both ends of the double-stranded cDNA, 3′-dA was added, and the index adapter was ligated. Adapter-ligated cDNA was amplified by PCR using a polymerase that did not selectively amplify dUTP, and the sequence library was prepared. Library quality was confirmed using an Agilent 2100 Bioanalyzer. Subsequently, cluster formation was analysed using cBot (Illumina, Inc.), and sequences were analysed using HiSeq 2500 (Illumina, Inc.). Sequence data were mapped onto the genomic sequence of *Fragaria vesca* ssp. *vesca* (assembly FraVeaHawaii_1.0, https://www.ncbi.nlm.nih.gov/genome/3314?genome_assembly_id=34435). Mapped read counts were normalized to obtain FPKMs. When the FPKM is 0, 0.0001 is added to the entire FPKM value to prevent calculation errors. Subsequently, logarithm values with two as the base were calculated from the expression level values, and the log expression values were used for comparisons.

### Expression analysis (real-time PCR)

Total RNA was purified from 10 strawberries using an ISOSPIN Plant RNA kit (Nippon Gene., LTD.). Then, cDNA was synthesized using a Maxima First Strand cDNA Synthesis Kit for RT-qPCR (Thermo Fisher Scientific, Inc., Waltham, MA, USA). These procedures were carried out following the kit protocols. Primers were designed with Primer-Blast using Primer3 and BLAST (https://www.ncbi.nlm.nih.gov/tools/primer-blast/index.cgi?LINK_LOC=BlastHome). Primer sequences are shown in Supplementary Data 6. Real-time PCR was performed with TB Green™ Premix Ex Taq™ II (Tli RNaseH Plus) (TaKaRa Bio Inc.). Quant studio 3 (Thermo Fisher Scientific, Inc.) and QuantStudio Design & Analysis software v1.4.1 were used. The elongation factor 1-alpha gene was used as the internal standard. The level of gene expression was determined using a relative standard curve. Significance levels between the *trans*-2-hexenal and control treatments were determined using t-tests.

### Quantification of JA in strawberry

In the manner described above, strawberries and 0.1 μmol or 1 μmol *trans*-2-hexenal were incubated in 120-mL plastic cups for 24 h. Hexane was used as a control. Strawberry’ JA levels were determined using a liquid chromatograph–tandem mass spectrometer (LC/MS/MS) system according to Ozawa *et al*.^29^, with minor modifications. Frozen strawberry (0.5 g) ground in liquid nitrogen were homogenized with ethyl acetate (2.5 mL), spiked with 10 ng of d2-JA (Tokyo Chemical Industries Co., Ltd., Tokyo, Japan) as an internal standard. After centrifugation at 5,000 rpm for 10 min at 4 °C, 1 mL of supernatant was transferred to a 1.5-mL tube and then evaporated to dryness under vacuum. The residue was suspended in 50 µL of 70% methanol/water (v/v) and centrifuged to clarify the liquid phase. The supernatant was analysed using an LC/MS/MS system (LCMS-8040, Shimadzu Co., Kyoto, Japan). Separation by HPLC was performed with a Mightysil RP-18 GP column (100 × 2.0 mm, 3-µm particle size; Kanto Chemical, Tokyo, Japan) at a flow rate of 200 µL/min. A linear gradient [0.1% formic acid aq. (A) and methanol (B), 5%–95% B/(A + B) for 16 min] was applied. Concentrations of JA and d2-JA were determined by multiple reaction monitoring. The monitored mass transitions were *m/z* 209 to *m/z* 59 for JA and *m/z* 211 to *m/z* 59 for d2-JA. The MS conditions were optimized for multiple reaction monitoring using authentic d2-JA (Tokyo Chemical Industries Co., Ltd.) and JA (Tokyo Chemical Industries Co., Ltd.) compounds. Bruised strawberries were excluded from the analysis. The numbers of strawberries used for the analysis were 9, 6, 8, and 6 for 0 (untreated), 0 (Hexane), 0.8, and 8 nmol/cm3 of *trans*-2-hexenal.

## Supplementary information

Supplementary information figure legend

Data set 1-10

## Data Availability

The datasets (SRA accession: PRJNA526582) analysed during the current study are available in the NCBI repository [https://www.ncbi.nlm.nih.gov/sra/PRJNA526582].
