# Supplementary material for: Effects of trans-2-hexenal and cis-3-hexenal on post-harvest strawberry
Source: Sci Rep. 2019 Jul 12;9:10112. doi: 10.1038/s41598-019-46307-4 (PMC6626038; doi:10.1038/s41598-019-46307-4)
Supplement: Supplementary file 1 — Supplementary information figure legend [file 41598_2019_46307_MOESM1_ESM.docx]

**Effects of trans-2-hexenal and cis-3-hexenal on post-harvest strawberry**

**Junko Wakai, Shoko Kusama, Kosuke Nakajima, Shikiho Kawai, Yasuaki Okumura, Kaori Shiojiri**

**Supplementary information**

Supplementary Data 1. Appearance of GLV-treated strawberries was not changed.

The same treated samples are shown (N = 5).

Supplementary Data 2. A 50 µmol/120 mL trans-2-hexenal-treated strawberry.

Supplementary Data 3. Botrytis cinerea-infected strawberry.

Supplementary Data 4. The quality of transcriptome sequencing.

Supplementary Data 5. The expression levels of 19 genes in 10 individual strawberries.

Supplementary Data 6. Primer sequences for RT-PCR.

Supplementary Data7. Sampling equipment to determine the acetate concentration released from strawberry.

Supplementary Data 8. The growth of Botrytis cinerea in the presence of green leaf volatiles (GLVs) was investigated.

Supplementary Data 9. Up-regulated gene list.

Supplementary Data 10. Down-regulated gene list.
