# Supplementary material for: Effects of trans-2-hexenal and cis-3-hexenal on post-harvest strawberry
Source: Sci Rep. 2019 Jul 12;9:10112. doi: 10.1038/s41598-019-46307-4 (PMC6626038; doi:10.1038/s41598-019-46307-4)
Supplement: Supplementary file 2 — Data set 1-10 [file 41598_2019_46307_MOESM2_ESM.pdf]

Effects of *trans*-2-hexenal and *cis*-3-hexenal on post-harvest strawberry

Junko Wakai, Shoko Kusama, Kosuke Nakajima, Shikiho Kawai, Yasuaki Okumura, Kaori Shiojiri

1 2 3 4 5

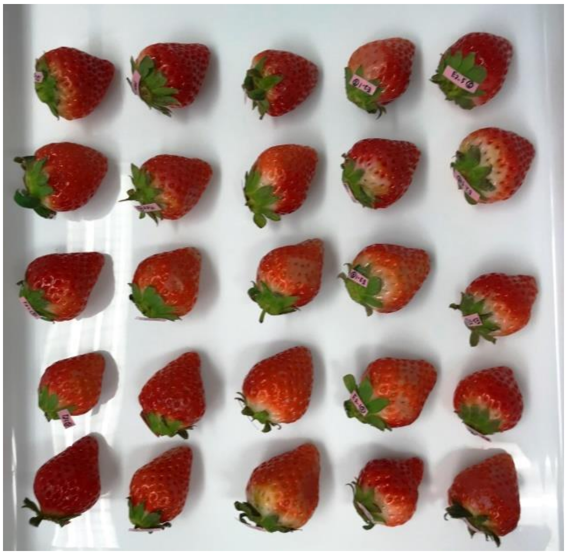

Supplementary Data 1

Appearance of GLV-treated strawberries was not changed.
The same treated samples are shown (N = 5).

Column 1: non-treated strawberry;
Column 2: hexane-treated strawberry;
Column 3: 0.1 µmol/120 mL *trans*-2-hexenal-treated strawberry;
Column 4: 1 µmol/120 mL *trans*-2-hexenal-treated strawberry;
Column 5: 5 µmol/120 mL *trans*-2-hexenal-treated strawberry

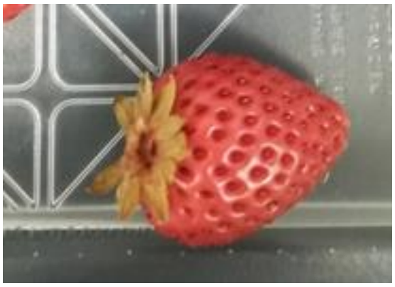

Supplementary Data 2

A 50 µmol/120 mL *trans*-2-hexenal-treated strawberry

Although the appearance was minimally changed, elasticity was lost and fruit were easily deformed by touch and presented tissue necrosis.

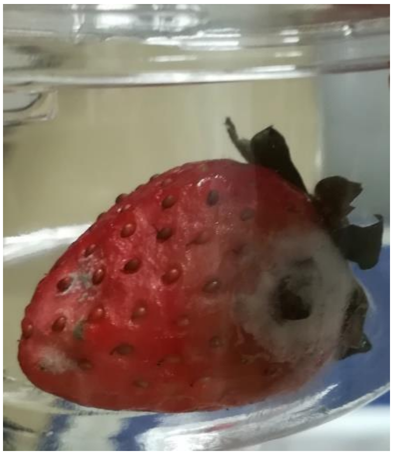

Supplementary Data 3

Botrytis cinerea-infected strawberry

Gray mold has spread and the fruit was classified as not alive.

Supplementary Data 4

The quality of transcriptome sequencing.
Sequencing quality scores measure the probability that a base is called incorrectly. QV30 represents an error rate of 1 in 1,000, with a corresponding call accuracy of 99.9% (https://www.illumina.com/science/education/sequencing-quality-scores.html).

Sample_title	Sample_name	Library ID	Read count (Read1 + Read2)	Number of bases (Read1 + Read2)	The ratio of the number of bases of QV 30 or more (Read1)	The ratio of the number of bases of QV 30 or more (Read2)	Description
Hexenal-Day0	PR0849_01_a	PR0849_01 A01_H1	63,095,510 (19.0%)	6,309,551,000	96.2	93.2	Strawberry fruit treated with *trans*-2-hexenal.
Control-Day0	PR0849_02_a	PR0849_02 A04_H1	49,990,738 (15.0%)	4,999,073,800	96.3	93.6	Strawberry fruit treated with hexane as control.

Supplementary Data 5

The expression levels of 19 genes in 10 individual strawberries.

a: aspartic proteinase PCS1
b: flavonoid 3'-monooxygenase-like
c: 12-oxophytodienoate reductase 3-like
d: uncharacterized LOC101308116
e: aminoacyl tRNA synthase complex-interacting multifunctional protein 1
f: palmitoyl-monogalactosyldiacylglycerol delta-7 desaturase, chloroplastic-like
g: putative disease resistance protein RGA3
h: putative laccase-1
i: probable serine/threonine-protein kinase Cx32, chloroplastic
j: uncharacterized LOC101294520
k: phospholipase D epsilon
l: proline-rich protein 36
m: phospholipase A1-Ibeta2, chloroplastic
n: probable receptor-like protein kinase At5g39020
o: gibberellin 2-beta-dioxygenase 2
p: probable receptor-like protein kinase At1g30570
q: nucleosome assembly protein 1;4-like
r: transcription initiation factor TFIID subunit 1-like
s: beta-fructofuranosidase, soluble isoenzyme I-like

Supplementary Data 5

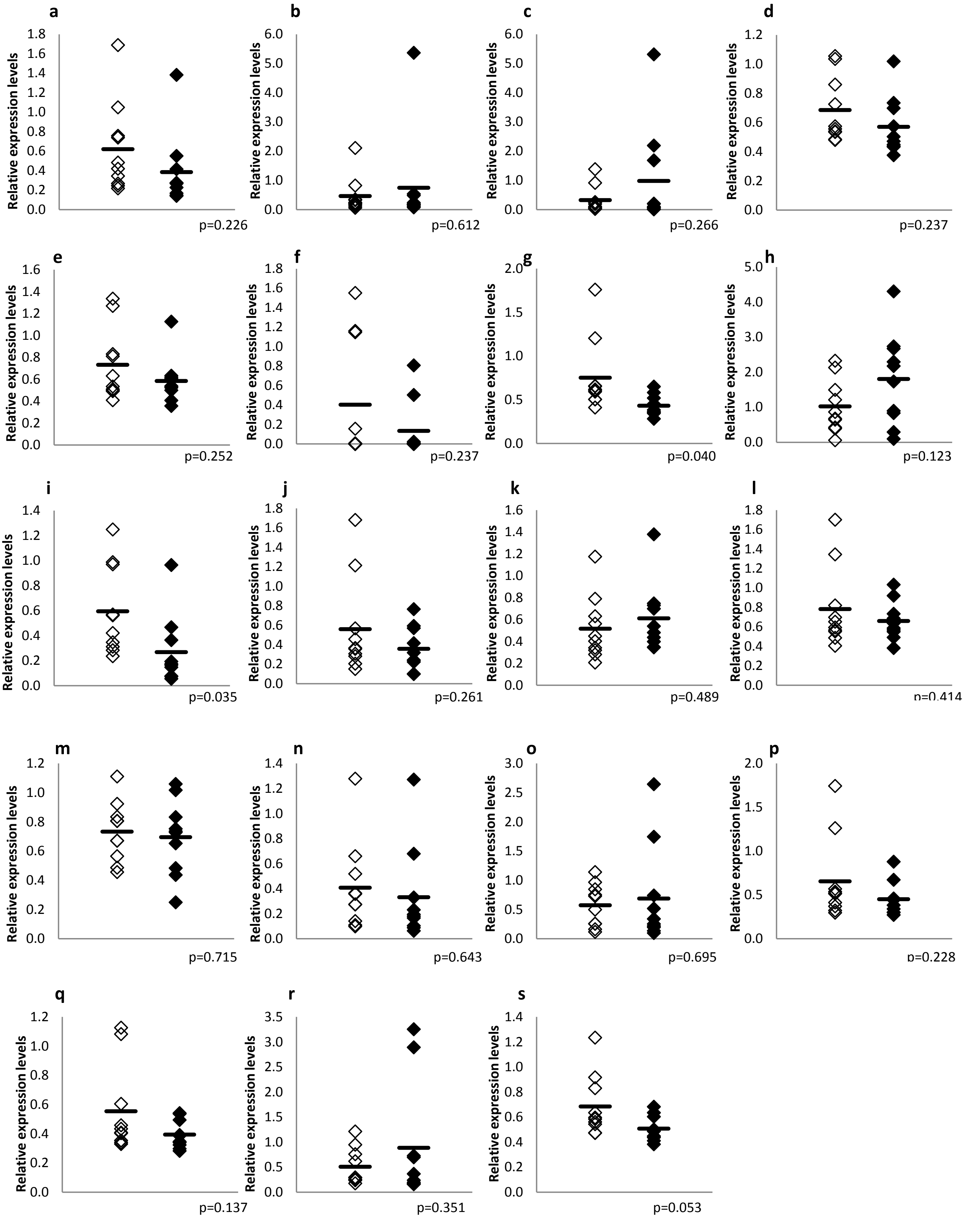

Supplementary Data 6

Primer sequences for RT-PCR.

Gene Name	Accession No.		Sequence
Sigma factor-binding protein 1, chloroplastic-like	XM_004308523.1	sense<br>anti-sense	5’-AGTTCAGGGCACTTGTGCAG-3’<br>5’-CACGATCGGCTTCCTTGAGT-3’
WRKY transcription factor 33	XM_004302509.2	sense<br>anti-sense	5’-CGACCGTTCAACTGAGCAAC-3’<br>5’-TAGGAACATGGGGGAGCTGA-3’
Universal stress protein A-like protein transcript variant X1	XM_004302198.2	sense<br>anti-sense	5’-GCACAGGTAGGAGTCAGAGTT-3’<br>5’-TCCTGAAGAACATCCCTGCAC-3’
Elongation factor 1-alpha	XM_004309832.2	sense<br>anti-sense	5’-TTCGCTCCCACTGGATTGAC-3’<br>5’-ACCCACGCTTGAGATCCTTG-3’
aspartic proteinase PCS1	XM_004302721.2	sense<br>anti-sense	TTGTGGGCCCATGCATCTT<br>GCTCTCACTACACATAAATTGGCA
flavonoid 3'-monooxygenase-like	NM_001287418.1	sense<br>anti-sense	TCCCGAAGGGTTCCACATTG<br>TTCGGGCCTGAACTCCAAC
12-oxophytodienoate reductase 3-like	XM_004287578.2	sense<br>anti-sense	ACTCATGATCCTGTTGTTGGGT<br>GAGCATCAAAACATAAGGGGAGC
uncharacterized LOC101308116	XM_004306054.2	sense<br>anti-sense	TTTTGCCCAAACTAGCTGGC<br>GGAGGTGTTACAGACATCAGGT
aminoacyl tRNA synthase complex-interacting multifunctional protein 1	XM_004287150.2	sense<br>anti-sense	GAAAAGGCGAGCAAGGTAGC<br>TTAGGAGACGGCCAGAGGAA
palmitoyl-monogalactosyldiacylglycerol delta-7 desaturase, chloroplastic-like	XM_004287869.2	sense<br>anti-sense	CAGTAGCATGGAGCAGGAGG<br>TCACTTTGGTTCAACACAGAAACA
putative disease resistance protein RGA3	XM_004305114.1	sense<br>anti-sense	GCGCATCTCTTCAGCAACTG<br>GTGCAGTACTGTAGCCCCTC
putative laccase-1	XM_011459691.1	sense<br>anti-sense	GTGGACCAACGTCTTTTGCC<br>CTTGGTGTTGCGGTCCTAGT
probable serine/threonine-protein kinase Cx32, chloroplastic	XM_004289189.2	sense<br>anti-sense	TCCGACTGAGGGCAGAAAAC<br>CGTCTGTCACGAGCTCTACC
uncharacterized LOC101294520	XM_004289764.2	sense<br>anti-sense	AGAATCGGTGCTCGTTGATCT<br>GTCCCCATAGTAGTATTCACTGGT
phospholipase D epsilon	XM_004297558.2	sense<br>anti-sense	GCTGACAACTTACAGTGGCAA<br>TCCTCCATGTTGATTTGTCTTCCT
proline-rich protein 36	XM_004299622.2	sense<br>anti-sense	CCACTCCCCAAAACCTCACT<br>CCCAGAAGAAACAGAGACCCTT
phospholipase A1-Ibeta2, chloroplastic	XM_004300296.2	sense<br>anti-sense	ACACATCTCCGTTGAACCCC<br>CTGCAGCCGATGATGCTTTAAT
probable receptor-like protein kinase At5g39020	XM_004308500.2	sense<br>anti-sense	TGCAGAGGCTATTGTGGTGT<br>GCAACATGTTCCGAGTCTAATCA
gibberellin 2-beta-dioxygenase 2	XM_004288310.2	sense<br>anti-sense	TTGATCTGGTCGGAGAGGGT<br>AAGTCCTTGTCCAGTGGTGG
probable receptor-like protein kinase At1g30570	XM_011462342.1	sense<br>anti-sense	TGGGGTTTCATGTTTTTAGCTCC<br>AGACCTCGATTGAGCTTCACA
nucleosome assembly protein 1;4-like	XM_011470222.1	sense<br>anti-sense	GCTCTAGAGAGCTTGGTTGGA<br>CTTCAGTGCAGCTCTCTCAAAAA
transcription initiation factor TFIID subunit 1-like	XM_011461870.1	sense<br>anti-sense	TTCAGAATCCAGTCACCTCGG<br>GATACCCCTCCAAGTTTGGTCA
beta-fructofuranosidase, soluble isoenzyme I-like	XM_004307757.2	sense<br>anti-sense	AGGAGTTGCATTCATCCGAGA<br>GAAGGGCATACGTACCCTGG

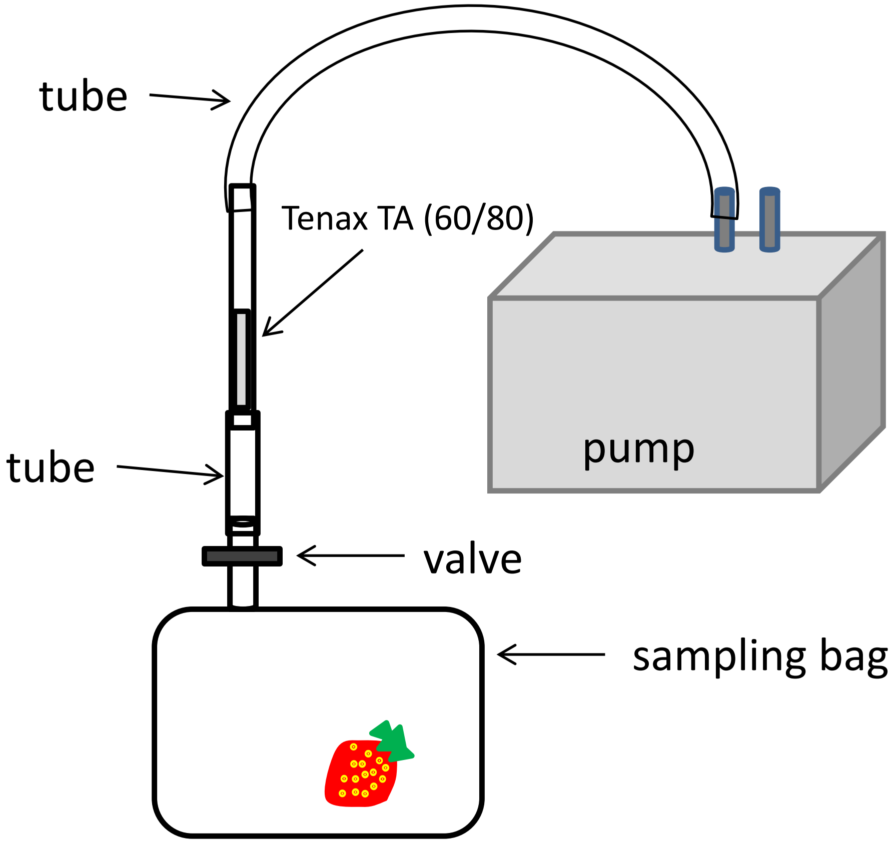

Supplementary Data 7

Sampling equipment to determine the acetate concentration released from strawberry.

Supplementary Data 8

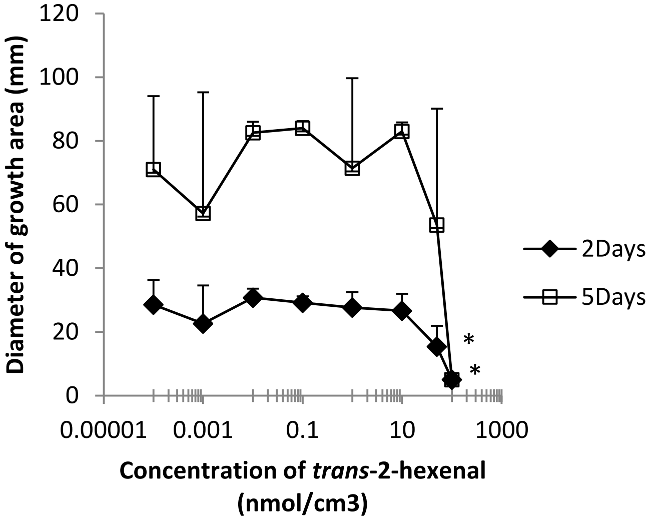

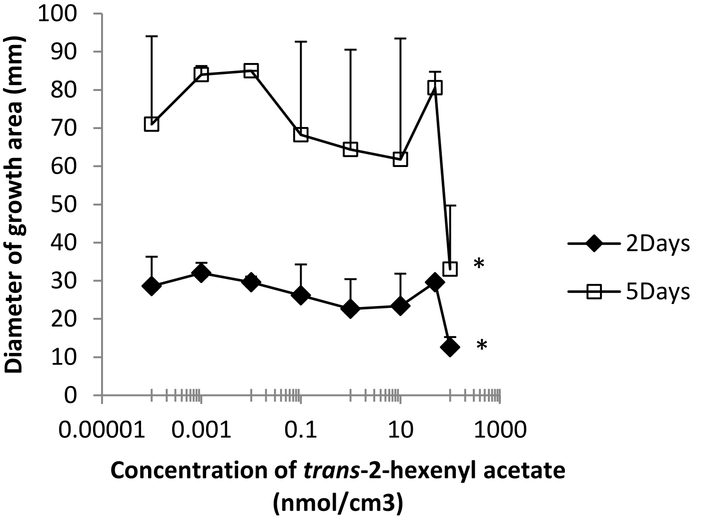

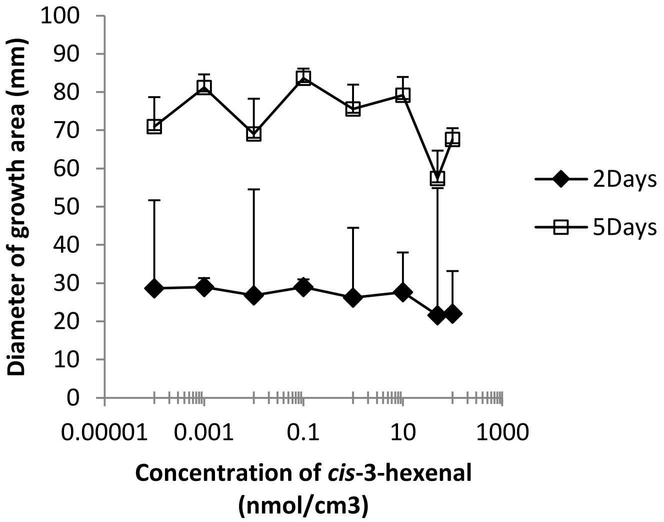

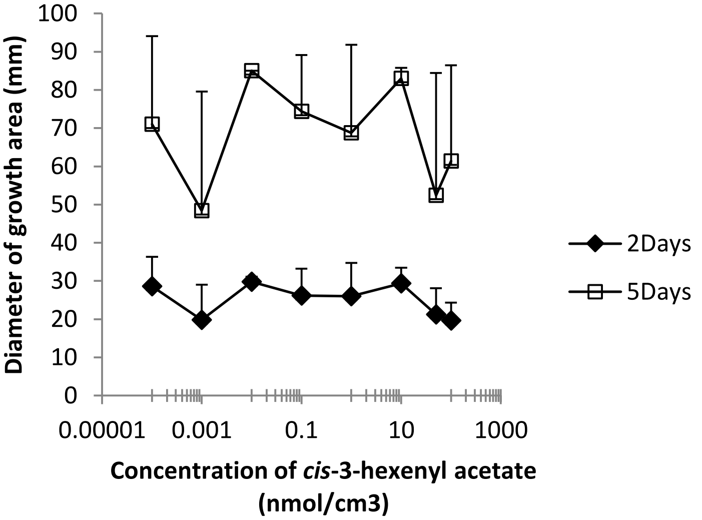

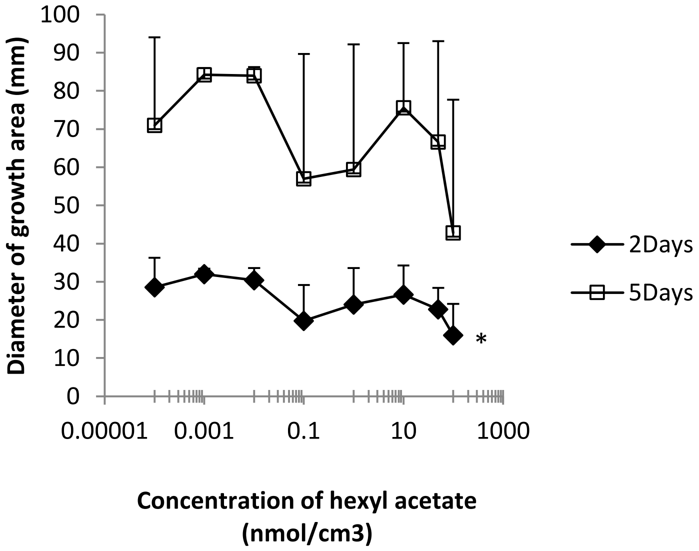

Supplementary Data 8

The growth of *Botrytis cinerea* in the presence of green leaf volatiles (GLVs) was investigated.

Botrytis cinerea was incubated on PDA medium with a GLV gas at 25° C. After 2 or 5 d, the diameters of the growth areas were measured. The averages of the PDA plates (N = 5) are plotted, and the error bars represent standard deviations. The following GLVs were used: *tran*s-2-hexenal, *cis*-3-hexenal, *trans*-2-hexenyl acetate, *cis*-3-hexenyl acetate, and hexyl acetate. The GLV concentrations were from 0.001 $nmol/cm^3$ to 100 $nmol/cm^3$. As a control, hexane, which was the diluting solvent, was used. In each graft, the 0.0001 $nmol/cm^3$ plot was used as the control.

* $P < 0.05$ versus control

Supplementary Data 9

Up-regulated gene list.

No.	Name	FPKM_log2 (Hexenal)	FPKM_log2 (Control)	ΔFPKM_log2	Transcript ID	Description
1	rpl36	7.41	-13.29	20.70	YP_004286081.1<br>YP_004286136.1	-
2	rps19	4.35	-13.29	17.64	YP_004286081.1<br>YP_004286142.1	-
3	rps16	3.12	-13.29	16.41	YP_004286084.1	-
4	LOC101292354	2.39	-13.29	15.68	XM_004308523.1	sigma factor binding protein 1, chloroplastic-like
5	LOC101302669	-0.86	-3.99	3.12	XM_004302721.2	aspartic proteinase PCS1
6	LOC101314754	-0.50	-3.56	3.06	NM_001287418.1	flavonoid 3'-monooxygenase-like
7	LOC101293036	-0.76	-3.40	2.64	XM_004287578.2	12-oxophytodienoate reductase 3-like
8	LOC101308116	0.25	-2.35	2.60	XM_004306054.2	uncharacterized LOC101308116
9	LOC101308900	0.55	-1.86	2.41	XM_004287150.2	aminoacyl tRNA synthase complex-interacting multifunctional protein 1
10	LOC101308020	-0.35	-2.72	2.37	XM_004287869.2	palmitoyl-monogalactosyldiacylglycerol delta-7 desaturase, chloroplastic-like
11	LOC101300362	-2.46	-4.81	2.36	XM_011468210.1<br>XM_004302633.2<br>XM_011468211.1<br>XM_011468209.1<br>XM_004305114.1	putative disease resistance protein RGA3
12	LOC101298863	-1.57	-3.75	2.18	XM_011459691.1	putative laccase-1
13	LOC101297590	-0.30	-2.45	2.15	XM_004289189.2	probable serine/threonine-protein kinase Cx32, chloroplastic
14	LOC101294520	0.53	-1.53	2.06	XM_004289764.2	uncharacterized LOC101294520
15	LOC101298884	-1.85	-3.86	2.01	XM_004297558.2	phospholipase D epsilon
16	LOC101301971	1.33	-0.54	1.87	XM_004299622.2	proline-rich protein 36
17	LOC101290867	-1.18	-2.87	1.69	XM_004300296.2	phospholipase A1-Ibeta2, chloroplastic
18	LOC101307840	-1.42	-3.11	1.69	XM_004308500.2	probable receptor-like protein kinase At5g39020
19	LOC101295656	-0.61	-2.28	1.67	XM_004288310.2	gibberellin 2-beta-dioxygenase 2
20	LOC101295118	-1.24	-2.90	1.66	XM_011462342.1	probable receptor-like protein kinase At1g30570
21	LOC101291157	3.99	2.33	1.66	XM_004302199.2<br>XM_004302198.2	universal stress protein A-like protein
22	LOC105352662	1.34	-0.31	1.65	XM_011470222.1<br>XM_011470223.1	nucleosome assembly protein 1;4-like
23	LOC105350238	-1.22	-2.85	1.63	XM_011461870.1	transcription initiation factor TFIID subunit 1-like
24	LOC101302788	0.34	-1.29	1.63	XM_004307757.2	beta-fructofuranosidase, soluble isoenzyme I-like
25	LOC101291251	0.52	-1.08	1.60	XM_004301786.1	F-box protein SKIP2-like
26	LOC101295075	-0.20	-1.80	1.60	XM_004287584.2	MOB kinase activator-like 1
27	LOC101311594	-0.54	-2.14	1.60	XM_004304155.2	truncated transcription factor CAULIFLOWER A-like
28	LOC101296840	-1.15	-2.75	1.60	XM_011461874.1	tobamovirus multiplication protein 1-like
29	LOC101295313	1.16	-0.39	1.55	XM_004294373.2	pirin-like protein
30	LOC101299792	0.38	-1.16	1.54	XM_011469837.1<br>XM_004304422.2	uncharacterized LOC101299792

Supplementary Data 9

Up-regulated gene list.

No.	Name	FPKM_log2 (Hexenal)	FPKM_log2 (Control)	ΔFPKM_log2	Transcript ID	Description
31	LOC101314223	0.39	-1.08	1.48	XM_011471725.1	superoxide dismutase [Fe], chloroplastic
32	LOC101295419	0.45	-0.96	1.41	XM_004299600.2	7-deoxyloganetin glucosyltransferase-like
33	LOC101299115	-0.40	-1.81	1.41	XM_004306029.2	transcription initiation factor IIB-2
34	LOC101312610	-1.04	-2.45	1.41	XM_011461954.1<br>XM_004293473.2	serine carboxypeptidase-like 31
35	LOC101304111	-1.62	-3.02	1.41	XM_011469201.1<br>XM_011469200.1<br>XM_011469199.1<br>XM_004303738.2	uncharacterized LOC101304111
36	LOC101294417	2.19	0.80	1.39	XM_004290324.2	39S ribosomal protein L41, mitochondrial-like
37	LOC101299491	0.09	-1.27	1.37	XM_004302148.2	two pore potassium channel a-like
38	LOC101309106	0.56	-0.80	1.36	XM_011459546.1<br>XM_011459541.1<br>XM_011459544.1<br>XM_011459548.1<br>XM_011459547.1<br>XM_011459545.1<br>XM_011459542.1<br>XM_011459543.1	LIM domain-containing protein WLIM1-like
39	LOC101310559	-0.53	-1.88	1.36	XM_004289480.2	uncharacterized LOC101310559
40	LOC101315234	-1.18	-2.54	1.36	XM_004298613.2	acid beta-fructofuranosidase-like
41	LOC101294168	-0.33	-1.66	1.34	XM_004297902.2	uncharacterized LOC101294168
42	LOC101303469	-0.54	-1.88	1.34	XM_004309241.2	TMV resistance protein N-like
43	LOC105349590	2.40	1.06	1.33	XM_011459566.1 :<br>XM_011459565.1	uncharacterized LOC105349590
44	LOC101312902	1.07	-0.26	1.33	XM_011461275.1	elongation of fatty acids protein 3-like
45	LOC101296759	-0.29	-1.62	1.33	XM_011462830.1 :<br>XM_011462831.1	G-type lectin S-receptor-like serine/threonine-protein kinase At4g27290
46	LOC101310901	-0.65	-1.97	1.32	XM_011465462.1	flavonoid 3',5'-hydroxylase-like
47	LOC101315295	-0.31	-1.62	1.32	XM_004289907.2	protein CUP-SHAPED COTYLEDON 3-like
48	LOC101312763	-0.52	-1.83	1.32	XM_004306771.2<br>XM_011470753.1<br>XM_011470752.1	uncharacterized LOC101312763
49	LOC101313200	-1.59	-2.90	1.32	XM_011463791.1	disease resistance protein At4g27190-like
50	LOC101300046	0.35	-0.94	1.29	XM_004297320.2	serine carboxypeptidase-like 50
51	LOC101302567	0.68	-0.60	1.28	XM_004302320.2<br>XM_011467951.1	probable mitochondrial chaperone BCS1-B
52	LOC101300084	-0.24	-1.52	1.28	XM_011468316.1<br>XM_004305185.2	F-box protein CPR30-like
53	LOC101304972	-0.86	-2.14	1.28	XM_004300501.2	dof zinc finger protein DOF3.7-like
54	LOC101303683	-1.12	-2.40	1.28	XM_004294705.2	F-box/kelch-repeat protein At3g06240-like
55	LOC105353149	-1.61	-2.88	1.28	XM_011471914.1	cyclic nucleotide-gated ion channel 1-like
56	LOC101292016	-2.04	-3.32	1.28	XM_011464729.1	alpha-1,4 glucan phosphorylase L-2 isozyme, chloroplastic/amyloplastic-like
57	LOC101303801	-0.97	-2.24	1.27	XM_011465384.1	uncharacterized LOC101303801
58	LOC101293810	-0.44	-1.70	1.26	XM_004305934.1	probable disease resistance protein At5g66900
59	LOC101300386	-1.05	-2.32	1.26	XM_004306891.2	probable pectinesterase/pectinesterase inhibitor 17
60	LOC101312813	0.10	-1.16	1.26	XM_004295403.2	reticuline oxidase-like protein

Supplementary Data 9

Up-regulated gene list.

No.	Name	FPKM_log2 (Hexenal)	FPKM_log2 (Control)	ΔFPKM_log2	Transcript ID	Description
61	LOC101311823	-0.55	-1.80	1.25	XM_004309962.2	very-long-chain 3-oxoacyl-CoA reductase 1-like
62	LOC105350861	-0.18	-1.43	1.25	XM_011463842.1	uncharacterized LOC105350861
63	LOC101313572	-0.09	-1.34	1.24	XM_004291168.2	dehydration-responsive protein RD22
64	LOC101299119	-0.31	-1.55	1.24	XM_011462543.1	uncharacterized LOC101299119
65	LOC101306051	0.17	-1.05	1.22	XM_004300582.2	glutathione S-transferase U17-like
66	LOC105353295	1.29	0.08	1.21	XM_011472321.1	uncharacterized LOC105353295
67	LOC101311970	1.01	-0.17	1.19	XM_011465858.1<br>XM_004300965.2	pachytene checkpoint protein 2 homolog
68	LOC101314296	0.91	-0.28	1.19	XM_004303616.2	eukaryotic initiation factor 4A-6-like
69	LOC101292176	0.31	-0.87	1.19	XM_004290776.2	major allergen Pru ar 1-like
70	LOC101294218	0.28	-0.91	1.19	XM_004307808.2	ribosome-binding protein 1
71	LOC105352787	-0.03	-1.22	1.19	XM_011470487.1	uncharacterized LOC105352787
72	LOC101292722	-0.17	-1.36	1.19	XM_011468685.1	uncharacterized LOC101292722
73	LOC101292249	-1.21	-2.40	1.19	XM_004287823.2	probable mitochondrial chaperone BCS1-B
74	LOC105350176	-1.33	-2.52	1.19	XM_011461637.1	putative receptor-like protein kinase At4g00960
75	LOC101304623	-1.59	-2.77	1.19	XM_004288912.2	uncharacterized LOC101304623
76	LOC101292407	-2.23	-3.41	1.19	XM_004298267.2<br>XM_011464646.1<br>XM_011464645.1	phosphoenolpyruvate carboxylase 4
77	LOC101310904	-0.05	-1.20	1.16	XM_011466362.1	sister chromatid cohesion 1 protein 1
78	LOC101294672	2.46	1.31	1.15	XM_004301972.2	uncharacterized LOC101294672
79	LOC101311306	0.52	-0.62	1.15	XM_004305494.2	histone-lysine N-methyltransferase ATXR4
80	LOC105351177	0.46	-0.68	1.14	XM_011464908.1	alpha-mannosidase-like
81	LOC101307618	0.11	-1.03	1.14	XM_004287309.2	early nodulin-93-like
82	LOC101299947	0.87	-0.27	1.13	XM_004296225.2	uncharacterized LOC101299947
83	LOC101312956	0.29	-0.84	1.13	XM_004287242.2	umecyanin-like
84	LOC105352100	0.23	-0.90	1.13	XM_011468163.1	uncharacterized LOC105352100
85	LOC101292731	0.08	-1.05	1.13	XM_011468391.1	dynein light chain 1, cytoplasmic-like
86	LOC101305654	-0.32	-1.45	1.13	XM_004297092.2	AP2/ERF and B3 domain-containing transcription repressor TEM1-like
87	LOC101314802	-0.64	-1.77	1.12	XM_011468480.1	protein MKS1
88	LOC101291621	1.74	0.63	1.12	XM_011465295.1	polyadenylate-binding protein 7-like
89	LOC101296788	0.30	-0.82	1.12	XM_011469034.1	cysteine-rich and transmembrane domain-containing protein A
90	LOC105352189	-0.24	-1.35	1.12	XM_011468559.1<br>XM_011468557.1<br>XM_011468558.1	probable phospholipid hydroperoxide glutathione peroxidase

Supplementary Data 9

Up-regulated gene list.

No.	Name	FPKM_log2 (Hexenal)	FPKM_log2 (Control)	ΔFPKM_log2	Transcript ID	Description
91	LOC101311096	-0.25	-1.37	1.12	XM_004300365.2	uncharacterized LOC101311096
92	LOC101311713	-0.32	-1.44	1.12	XM_011470909.1	enhancer of mRNA-decapping protein 4-like
93	LOC101290736	-0.16	-1.26	1.10	XM_004293802.2	peptidyl-prolyl cis-trans isomerase FKBP13, chloroplastic
94	LOC101291663	-0.38	-1.48	1.10	XM_011464405.1<br>XM_004287821.2	putative F-box protein PP2-B12
95	LOC101301108	-1.30	-2.40	1.10	XM_004299860.2	MLO-like protein 4
96	LOC101291535	-1.73	-2.83	1.10	XM_011465869.1<br>XM_004300974.2	cytochrome P450 71A3-like
97	LOC101310229	1.14	0.04	1.10	XM_004301180.2	cold-inducible RNA-binding protein
98	LOC101297033	-0.18	-1.28	1.09	XM_004292220.1	probable disease resistance protein At5g66900
99	LOC101313631	-0.59	-1.69	1.09	XM_011469460.1<br>XM_011469459.1	uncharacterized LOC101313631
100	LOC101296477	0.83	-0.25	1.08	XM_004296527.2	1,2-dihydroxy-3-keto-5-methylthiopentene dioxygenase 1
101	LOC105350441	-0.35	-1.42	1.08	XM_011462467.1<br>XM_011462466.1	uncharacterized protein At5g64816-like
102	LOC101309207	-0.19	-1.25	1.06	XM_004290363.2	transcription factor ORG2-like
103	LOC101303684	2.34	1.28	1.05	XM_011463920.1	uncharacterized LOC101303684
104	LOC101296990	1.35	0.30	1.05	XM_011468196.1	uncharacterized LOC101296990
105	LOC101303696	0.62	-0.43	1.04	XM_004297249.2<br>XM_011464766.1	50S ribosomal protein L18, chloroplastic
106	LOC101305410	0.72	-0.32	1.04	XM_004288987.1	F-box protein SKIP23-like
107	LOC101297490	-0.03	-1.07	1.04	XM_011469829.1	uncharacterized LOC101297490
108	LOC101314966	-0.75	-1.79	1.04	XM_004303237.2	abhydrolase domain-containing protein 8
109	LOC101305608	-0.91	-1.95	1.04	XM_011470576.1<br>XM_004308221.2	NAC domain-containing protein 89-like
110	LOC101298241	-1.00	-2.04	1.04	XM_011468407.1<br>XM_004305257.2	coatomer subunit beta'-1-like
111	LOC101298240	-1.98	-3.03	1.04	XM_004305033.2<br>XM_011468115.1	probably inactive leucine-rich repeat receptor-like protein kinase At3g28040
112	LOC105350609	0.77	-0.26	1.03	XM_011463123.1<br>XM_011463122.1	uncharacterized LOC105350609
113	LOC101302804	0.95	-0.07	1.02	XM_004309877.2	F-box protein At3g07870-like
114	LOC101295803	-0.97	-1.98	1.02	XM_004297782.2<br>XM_011463976.1	serine/threonine-protein kinase Aurora-3-like
115	LOC101304477	-1.06	-2.07	1.02	XM_004298660.2	ACT domain-containing protein ACR8-like
116	LOC101315017	-1.44	-2.46	1.02	XM_004309972.2	uncharacterized LOC101315017
117	LOC101294835	-1.50	-2.51	1.02	XM_011463690.1	probable leucine-rich repeat receptor-like serine/threonine-protein kinase At3g14840
118	LOC101306321	-1.51	-2.53	1.02	XM_011463223.1<br>XM_011463221.1<br>XM_011463222.1<br>XM_011463220.1<br>XM_004296045.2	F-box/kelch-repeat protein At3g23880-like

Supplementary Data 9

Up-regulated gene list.

No.	Name	FPKM_log2 (Hexenal)	FPKM_log2 (Control)	ΔFPKM_log2	Transcript ID	Description
119	LOC101290991	-1.67	-2.69	1.02	XM_011464516.1<br>XM_011464484.1<br>XM_011464444.1<br>XM_011464462.1	F-box/LRR-repeat protein At4g14103-like
120	LOC101295360	-2.36	-3.38	1.02	XM_011469407.1<br>XM_004305873.2	sister chromatid cohesion 1 protein 2-like
121	LOC101302126	0.97	-0.04	1.01	XM_004309046.2	uncharacterized LOC101302126
122	LOC101304836	0.51	-0.49	1.01	XM_004292055.2	uncharacterized LOC101304836
123	LOC101291185	-0.05	-1.05	1.00	XM_004308113.2	putative DNA-binding protein ESCAROLA

Supplementary Data 10

Down-regulated gene list.

No.	Name	FPKM_log2 (Hexenal)	FPKM_log2 (Control)	ΔFPKM_log2	Transcript ID	Description
1	LOC101301085	-0.01	0.99	-1.00	XM_004294698.2	peroxidase 4-like
2	LOC101300758	0.72	1.72	-1.00	XM_004287768.2<br>XM_011464013.1<br>XM_011464017.1	troponin I-like
3	LOC101310485	-2.18	-1.18	-1.01	XM_004291080.2	patatin-like protein 2
4	LOC105351079	-1.76	-0.75	-1.01	XM_011464501.1	uncharacterized LOC105351079
5	LOC101307348	-1.08	-0.07	-1.01	XM_011470585.1	protein YLS9
6	LOC101300532	-0.10	0.92	-1.01	XM_004298293.2	uncharacterized protein At1g04910-like
7	LOC101297649	0.72	1.74	-1.02	XM_004302546.2	GDSL esterase/lipase CPRD49-like
8	LOC101294496	-1.73	-0.71	-1.03	XM_011469065.1	cytokinin dehydrogenase 1
9	LOC101302987	2.31	3.35	-1.04	XM_004308069.2	dehydration-responsive element-binding protein 1B-like
10	LOC101295772	-1.57	-0.51	-1.06	XM_004290703.2<br>XM_011460394.1	uncharacterized LOC101295772
11	LOC101302662	-0.23	0.83	-1.07	XM_004301567.2	psbP domain-containing protein 3, chloroplastic
12	LOC101310454	-0.30	0.76	-1.07	XM_004288194.2<br>XM_004288195.2<br>XM_011459306.1	F-box/kelch-repeat protein At3g06240-like
13	LOC101293178	-1.11	-0.04	-1.07	XM_004295286.2	WEB family protein At1g12150
14	LOC105350063	0.90	1.97	-1.07	XM_011459817.1<br>XM_011461415.1	uncharacterized protein At4g08330, chloroplastic-like
15	LOC101300343	0.98	2.05	-1.07	XM_004298732.2	protein P21-like
16	LOC101293065	-2.93	-1.85	-1.08	XM_011459425.1<br>XM_011459420.1<br>XM_004291967.2<br>XM_011459424.1<br>XM_011459423.1<br>XM_011459422.1<br>XM_011459421.1	uncharacterized LOC101293065
17	LOC101295078	-1.86	-0.78	-1.08	XM_004307103.2	phytol kinase 1, chloroplastic
18	LOC101313628	-1.57	-0.49	-1.08	XM_011468466.1<br>XM_011468467.1<br>XM_004305297.2	protein ABSCISIC ACID-INSENSITIVE 5
19	LOC101297388	0.25	1.33	-1.08	XM_004289114.1	dirigent protein 15-like
20	LOC101313356	0.61	1.70	-1.09	XM_004307711.2	probable non-specific lipid-transfer protein AKCS9
21	LOC101307953	-0.04	1.06	-1.10	XM_011460639.1<br>XM_011460638.1	B3 domain-containing protein At5g42700-like
22	LOC101297472	-1.85	-0.74	-1.11	XM_004305598.2	protein trichome birefringence-like 8
23	ndhJ	0.43	1.54	-1.11	YP_004286105.1	-
24	LOC101299558	-0.20	0.92	-1.11	XM_004294071.2	epidermis-specific secreted glycoprotein EP1-like
25	LOC105350224	-2.38	-1.24	-1.14	XM_011461843.1<br>XM_011461842.1<br>XM_011461841.1	G-type lectin S-receptor-like serine/threonine-protein kinase At4g03230
26	LOC101306554	-2.01	-0.88	-1.14	XM_004306335.2	receptor-like serine/threonine-protein kinase SD1-8
27	LOC101313179	-1.95	-0.81	-1.14	XM_011459134.1	probable LRR receptor-like serine/threonine-protein kinase At1g67720
28	LOC101291424	-0.17	0.97	-1.14	XM_004297541.2<br>XM_011465014.1<br>XM_011465013.1	general transcription factor 3C polypeptide 6-like
29	LOC101298787	0.16	1.29	-1.14	XM_004296533.2	early light-induced protein 1, chloroplastic-like
30	LOC101300091	1.89	3.03	-1.14	XM_004287681.2<br>XM_011463362.1<br>XM_011463365.1	ankyrin repeat-containing protein At3g12360-like

Supplementary Data 10

Down-regulated gene list.

No.	Name	FPKM_log2 (Hexenal)	FPKM_log2 (Control)	ΔFPKM_log2	Transcript ID	Description
31	LOC101306788	-0.99	0.16	-1.15	XM_004292121.2	subtilisin-like protease
32	LOC101305301	-1.79	-0.64	-1.15	XM_004288022.2	probable disease resistance protein At5g66900
33	LOC101292649	-2.22	-1.05	-1.16	XM_004289989.2	lysine histidine transporter-like 8
34	LOC101300153	-1.51	-0.32	-1.19	XM_004301069.1	ABC transporter F family member 4-like
35	LOC101314994	-1.91	-0.72	-1.19	XM_004307476.2	heat shock cognate 70 kDa protein-like
36	LOC101305644	-2.20	-0.99	-1.21	XM_011463656.1	G-type lectin S-receptor-like serine/threonine-protein kinase At4g27290
37	LOC101291694	4.60	5.83	-1.23	XM_004291804.2	jasmonate O-methyltransferase-like
38	LOC101293199	-1.55	-0.31	-1.23	XM_004299034.2	3-ketoacyl-CoA synthase 6
39	LOC101307675	-1.27	-0.01	-1.26	XM_004294249.2	gibberellin 2-beta-dioxygenase 8
40	LOC101296188	-1.95	-0.69	-1.26	XM_004294611.2	ribulose bisphosphate carboxylase/oxygenase activase, chloroplastic-like
41	LOC101291987	-3.24	-1.98	-1.26	XM_011460921.1	pleiotropic drug resistance protein 1-like
42	LOC101312814	-1.76	-0.50	-1.26	XM_004295602.2	lysosomal Pro-X carboxypeptidase-like
43	LOC101297067	-1.33	-0.07	-1.26	XM_004302544.2	probable WRKY transcription factor 70
44	LOC101306798	0.16	1.44	-1.28	XM_004294246.2	protein PSY3-like
45	LOC101305273	-0.57	0.74	-1.31	XM_011466725.1<br>XM_004301574.2	peptidyl-prolyl cis-trans isomerase FKBP18, chloroplastic
46	LOC101301714	-2.13	-0.80	-1.32	XM_004287445.2	uncharacterized LOC101301714
47	LOC101308290	-2.38	-1.05	-1.33	XM_011465766.1	MATE efflux family protein DTX1-like
48	LOC101303995	-2.30	-0.90	-1.40	XM_004298303.2	cytochrome P450 CYP749A22-like
49	LOC101298326	-1.76	-0.36	-1.40	XM_004301355.2	F-box protein At5g07610-like
50	LOC101303655	-0.83	0.57	-1.40	XM_004289565.2	glycine-rich protein DOT1-like
51	LOC101309578	-0.34	1.10	-1.44	XM_004287712.2	thiosulfate sulfurtransferase 16, chloroplastic-like
52	LOC101310339	-3.94	-2.45	-1.48	XM_011467956.1	ABC transporter B family member 15-like
53	LOC101294980	-3.06	-1.58	-1.49	XM_011470776.1<br>XM_004306795.2	uncharacterized LOC101294980
54	LOC101294725	0.77	2.27	-1.50	XM_004291515.2	two-pore potassium channel 1-like
55	LOC101297334	-2.99	-1.48	-1.52	XM_004295222.2	uncharacterized LOC101297334
56	LOC101306038	-2.94	-1.39	-1.55	XM_004297932.2	putative calcium-transporting ATPase 13, plasma membrane-type
57	LOC101299871	-1.85	-0.27	-1.58	XM_011466013.1<br>XM_011466012.1<br>XM_011466010.1<br>XM_011466011.1	respiratory burst oxidase homolog protein A-like
58	LOC101306460	-0.53	1.08	-1.60	XM_004306679.2	uncharacterized LOC101306460
59	LOC101300494	-0.75	0.88	-1.64	XM_004290562.2	late embryogenesis abundant protein D-34
60	LOC101298438	-1.69	0.03	-1.72	XM_011468710.1	shikimate O-hydroxycinnamoyltransferase
61	LOC101310331	-2.09	-0.34	-1.75	XM_004303073.2	cytochrome P450 CYP749A22-like
62	LOC101297030	-1.70	0.36	-2.06	XM_004310072.2	somatic embryogenesis receptor kinase 1-like
63	LOC101309604	-2.20	0.04	-2.25	XM_004291077.2	uncharacterized LOC101309604
64	LOC101305911	-2.83	-0.55	-2.27	XM_011461017.1<br>XM_011461016.1	ankyrin repeat-containing protein At5g02620-like
65	LOC101300651	-2.24	0.58	-2.81	XM_004304276.2	uncharacterized LOC101300651
66	LOC101301717	-2.97	-0.07	-2.90	XM_004306662.2	basic endochitinase-like
67	psbL	-13.29	7.62	-20.91	YP_004286117.1	-
